# Antifungal Activity and Plant Growth-Promoting Properties of *Bacillus mojovensis* B1302 against Rhizoctonia Cerealis

**DOI:** 10.3390/microorganisms10081682

**Published:** 2022-08-20

**Authors:** Yanjie Yi, Pengyu Luan, Kang Wang, Guiling Li, Yanan Yin, Yanhui Yang, Qingyao Zhang, Yang Liu

**Affiliations:** 1 School of Biological Engineering, Henan University of Technology, Zhengzhou 450001, China; 2 The Key Laboratory of Functional Molecules for Biomedical Research, Zhengzhou 450001, China; 3 Henan Provincial Key Laboratory of Biological Processing and Nutritional Function of Wheat, Zhengzhou 450001, China

**Keywords:** *Bacillus mojovensis* B1302, *Rhizoctonia cerealis*, antifungal activity, plant growth-promotion, animal toxicological safety

## Abstract

*Rhizoctonia cerealis* is a worldwide soil-borne pathogenic fungus that significantly infects wheat and causes sharp eyespot in China. However, the biocontrol strains used for the control of *Rhizoctonia cerealis* are insufficient. In the present study, antagonistic strain B1302 from the rhizosphere of wheat were isolated and identified as *Bacillus mojovensis* based on their morphological, physiological, and biochemical characteristics, and their 16S rDNA sequence. Culture filtrate of strain B1302 had a broad antifungal spectrum. In order to improve the antifungal activity of B1302, response surface methodology (RSM) was used to optimize the culture conditions. The final medium composition and culture conditions were 13.2 g/L of wheat bran, 14.1 g/L of soybean meal, 224 r/min of rotation speed, 7.50 of initial pH, and 1.5 × 10^8^ CFU/mL of inoculation amount at 35 °C for a culture duration of 72 h. *B. mojavensis* B1302 inhibited the hyphae growth of *R.* *cerealis* and produced hydrolytic enzymes (protease, chitinase, and glucanase), IAA, and had N-fixing potentiality and P-solubilisation capacity. It can also promote wheat seedling growth in potted plants. The disease incidence and index of wheat seedlings were consistent with the effect of commercial pesticides under treatment with culture filtrate. The biocontrol efficacy of culture filtrate was significant—up to 65.25%. An animal toxicological safety analysis suggested that culture filtrate was safe for use and could be developed into an effective microbial fungicide to control wheat sharp eyespot.

## 1. Introduction

Wheat (*Triticum aestivum* L.), the third most important food crop, is widely planted around the world [1]. With downward economic pressure and the impact of COVID-19, the global demand for wheat will increase with the increase of environmental pressure [2,3]. Wheat production is seriously affected by various abiotic and biotic stresses. Among plant fungal diseases, wheat sharp eyespot is one of the most destructive diseases that affects wheat growth and yield and grain quality [4]. The soil-borne fungus, *R. cerealis,* is a major causal pathogen of wheat sharp eyespot. *R. cerealis* doesn’t produce asexual spores, so it exists in the soil as either a vegetative hyphae or sexual sclerotia [5]. The pathogen can enter the plant system through basal stems and basal sheaths, penetrate the cell wall, and eventually reach the interior of the cell to destroy the plants [6]. The transport system in wheat susceptible plants is impeded and they can be infected by the pathogen at all stages of growth and development [7]. Therefore, the fungus causes significant damage to the wheat and is a threat to world wheat production.

In China, chemical pesticides and field management are widely used in agriculture and forestry to control diseases. However, due to the pathogen’s natural persistence and various environmental factors in field management, the prevention and control effects are not ideal [8]. Many chemical pesticides, such as diniconazole, carbendazim, and thiophanate, can effectively reduce the occurrence of wheat sharp eyespot [9]. However, environmental pollution and resistant microbial strains induced by the overuse of chemical pesticides are growing problems. In response to this demand, biological control has become widely used as an environmentally-friendly and species-specific method for fungi disease control [10].

In recent years, many antagonistic microorganisms have been applied in the biocontrol of fungi diseases of agricultural plants [11]. More and more microbial strains are continuously being screened and isolated. Bacterial genera, such as *Bacillus*, *Streptomyces,* and *Pseudomonas,* are often used for the biocontrol of some fungal diseases [12,13,14]. Several reports demonstrate that some of the bacteria can also promote plant growth [15]. Research of *Bacillus* agents has shown that they resist fungal pathogens through antagonism, niche space and nutrients competition, and host resistance induction [16,17]. The biocontrol effects of certain strains are mainly relevant to their production of cyclic lipopeptides (CLPs), which exhibit excellent antifungal properties [18,19]. *B. circulans*, *B. sphaericus*, *B. subtilis*, *B. licheniformis*, etc. also have been used as biological control agents against different fungi diseases for a long time [20]. However, the number of *B. mojavensis* bacteria screened and isolated was less than the number of *B. subtilis*. According to the relevant literature, *B. mojavensis* can produce VOCs that act as plant growth modulators [21]. *B. mojavensis* has been reported to decrease fumonisins accumulation in maize [22]. Moreover, *B. mojavensis* can also produce cyclic lipopeptides (CLPs), which include iturin, surfactin, and fengycin [23,24]. Therefore, it is necessary to isolate and characterize different biocontrol strains.

This study aims to isolate and identify the antagonistic strains against *R. cerealis* and optimize the culture conditions for improving its antifungal activity. The antifungal spectrum, active substances, growth promotion, control efficacy, and animal safety were further analyzed, which laid the foundation for the development of effective biological fungicides against wheat sharp eyespot.

## 2. Materials and Methods

### 2.1. Materials

Soil samples were collected from the rhizosphere soil of wheat in Zhengzhou, Henan. Soil was loamy cinnamon soil (according to Chinese soil classification) with a pH of 7.9, an organic matter content of 1.21%, and a nutrient content of available N of 0.078%, available P of 7 ppm, and available K of 215 ppm. (www.soil.csdb.cn, accessed on 16 June 2021). A five-point sampling method was used to collect the rhizosphere soil samples (0–20 cm). After the wheat were dug out, the roots were carefully shaken to remove the loosely adhering soil, and the remaining attached soil was carefully collected as rhizosphere soil using a sterile brush.

Six plant pathogens, Rhizoctonia cerealis, Fusarium graminearum, Bipolaris sorokiniana, Alternaria solani, Bipolaris maydis Shoem, and Fusarium oxysporum, were stored at −20 °C in our laboratory. Pathogens were cultured on PDA medium (20 g of glucose, 200 g of potato, 15 g of agar, 1000 mL of sterile water). The culture conditions followed the methods of Min-Jeong Kim with appropriate modifications [16].

Wheat seed (Yumai 49) was provided by Henan Academy of Agricultural Sciences, which was widely planted in the Huang-Huai wheat production area.

KM mice (Certificate of conformity: SCXK 2015-0004) were purchased from the Henan experimental animal center with weights between 18–22 g, SPF grade, and in good health.

### 2.2. Isolation and Screening of the Antagonistic Bacteria

Bacteria were isolated from soil by serial dilution method. Every soil sample was serially diluted up to 10^−^^8^ with sterile distilled water. Subsequently, 100 μL of the diluted soil resuspension was spread on Luria–Bertani (LB) medium (5 g of yeast extract, 10 g of peptone, 10 g of NaCl, 1 L of sterile water, pH 7) plates and incubated at 37 °C for 48 h. The colonies with different morphologies were selected for purifying and testing the antifungal activity by the plate confrontation method [25]. Antagonistic strains inoculated into LB medium were cultured under agitation at 180 r/min at 37 °C for 2 days. Then, culture filtrate was prepared by filtering the supernatant of the culture through a 0.22-µm filter. The mycelial growth rate method was used to determine the inhibition rate of B1302 culture filtrate [26]. LB plates without culture filtrate were taken as the control. The process was repeated three times. The formula for the inhibition rate is as follows:

Inhibition rate = [(A − B) ÷ A] × 100%

where A is the mycelial radial diameter of the pathogen in the control plate and B is the mycelial radial growth diameter of the pathogen in the treatment group.

### 2.3. Identification of the Antagonistic Strain B1302

Strain B1302 was cultured on Luria–Bertani (LB) medium (5 g of yeast extract, 10 g of peptone, 10 g of NaCl, 1 L of sterile water, pH 7) at 37 °C for two days. A morphological, physiological, and biochemical analysis were performed, including colony morphology, size, color, transparency, carbon source utilization, gelatin lignification, starch hydrolysis, glucose consumption, oxidase reactions, and nitrate reduction [27].

A sequence analysis of 16S rDNA was used for molecular identification. Genomic DNA was extracted from the strain according to the method of Zakry et al. [28]. The 16S rDNA sequence was amplified by PCR using the universal primers, 16SF (5′-AGAGTTTGATCATGGCTCAG-3′) and 16SR (5′-ACGGTTACCTTGTTACGACTT-3′) and sequenced by the Sangon Biotech Co., Ltd. (Shanghai, China) [29]. Then, the sequence was analyzed through the BLAST tool on NCBI website (Bethesda, MD, USA; http://www.ncbi.nlm.nih.gov/Blast, accessed on 29 July 2021) and the nearest neighbor of the 16S rDNA sequence was determined. A phylogenetic tree was constructed by the neighbor-joining tree algorithm using a bootstrap value of the MEGA7 [30].

### 2.4. Detection of Antimicrobial Spectrum for B1302

The antagonistic activity of *B. mojavensis* B1302 culture filtrate towards different pathogens was tested using the mycelial growth rate method, as described previously [26]. The test was repeated three times.

### 2.5. Culture Conditions

A single factor method was taken to determine the optimal components of medium. The basic cultural conditions were 30 °C, 1% of inoculum, pH 7, 180 rpm, and 72 h. Various media with different components were used to test the optimal media and culture conditions for improving the antifungal activity. LB medium was supplemented with different carbon, bran density, nitrogen sources, soybean meal concentration, and inoculum amount, after which seed liquid was inoculated into the medium and incubated on a shaker at 180 rpm at 28 °C for 12 h. The effects of various pHs (6.0, 6.5, 7.0, 7.5, and 8.0) and cultivation times (48, 60, 72, 84, and 96 h) on the inhibition rate were then evaluated [27]. The experiment was repeated three times.

### 2.6. Selection of Significant Variables for Culture Filtrate Antifungal Activity by Plackett–Burman Design

The Plackett–Burman statistical design is an efficient method for screening and identifying the significant variables among a large number of variables that have significant effects on the process [31,32]. The main factors were screened from the seven factors (wheat bran, soybean meal, pH, temperature, speed, inoculation amount, and cultivation time) that influenced the fermentation culture conditions when the inhibition rate (Y) was taken as the response value. For screening out the most important factors as soon as possible, each factor took two levels, the level of ‘1′ and ‘−1′. The test factors and levels are shown in Table 1. The process was repeated three times.

### 2.7. Optimization of the Selected Significant Variables by Box–Behnken Design

The Plackett–Burman test was designed to identify three main factors affecting the culture conditions of antagonistic bacteria B1302. The Box–Behnken test design was applied to the 3 factors for 3 different levels (high (+ 1), medium (0), low (− 1)) for the response surface test, including 5 central tests and 12 factorial experiments [33]. The test factors and levels are shown in Table 2. Design-Expert 8.0.6 (Stat-Ease, Inc., Minneapolis, MN, USA) was used to analyze the regression equation of the response values of the 17 test points to obtain the optimal culture conditions for the antagonistic bacteria B1302. The process was repeated three times.

### 2.8. Antagonistic Activity of B1302 Culture Filtrates

The effect of *B. mojavensis* B1302 culture filtrates on the hyphal growth of *R. cerealis* was observed using optical microscopy. After pathogens were cultured in PDA medium with culture filtrates at 27–28 °C for 7–10 days, the hyphae were observed and recorded at a magnification of 10 × 20.

Antifungal activities against pathogens were performed by the mycelial growth rate method, as described previously. The final concentrations of B1302 culture filtrates ranged from 10%, 15%, 20%, 25%, 40%, 50%, 75%, and 100% using the LB culture dilution. The LB culture medium without culture filtrate was set as the control. After inoculation of the fungal mycelia onto the center of the solid medium, the dishes were incubated in the dark at 28 ± 0.5 °C. The experiment was repeated three times. When the fungal mycelium reached the edges in the control plates, the antifungal activities were calculated.

### 2.9. Extracellular Enzyme Activity and Assays for Detection of PGP Abilities

The enzyme activity was determined by the DNS method according to Nongnat et al., with some modifications, with glucose being a standard for the calibration curves [34]. Chitinase and β-1,3-glucanase activity was quantified and calculated in three repetitions.

The protease activity was determined using a modified Folin method [35]. To measure protease production in the culture filtrate, tyrosine was taken as the standard and repeated three times. The amount of enzyme required to produce an equivalent of 1 g of tyrosine per minute is defined as an enzyme viability unit (U).

The ability of the strain to grow in nitrogen-deficient media was detected using Ashby’s N-free mannitol agar [36]. A 200-μL aliquot of fresh culture was inoculated on the agar medium and cultured at 28 °C for 48 h. The development of visible growth on the plates was observed. The P concentrations were measured using the molybdenum antimony colorimetric method [37]. The culture filtrate was incubated under agitation at 180 r/min at 28 °C for 4 days, followed by centrifugation at 10,000 r/min for 15 min at 4°C. After reaction with molybdenum-antimony solution, the P concentration was calorimetrically determined at 700 nm. Indole-3-acetic acid from the strain was determined by the colorimetric method using Salkowski reagent [38]. The tests were repeated three times.

### 2.10. Evaluation of B. mojavensis B1302 Culture Filtrate on Wheat Growth

Strain B1302 was cultured in LB medium for 48 h, then centrifuged at 4000 rpm and resuspended with sterile water to adjust the final cell concentration to 1 × 10^8^ CFU/mL. Seeds of Yumai 49 soaked with different liquids (culture filtrate, fungicides, and sterile water) were planted in three pots, respectively. Then, the plants were grown in the same light intensity (4000 lx) and at a light:dark (L:D) ratio of 14 h:10 h. The triadimefon, carbendazim, *Validamycin·Bacillus,* and sterile water were set as positive and negative controls, respectively. The germination rate, height, and fresh weights of seedlings and roots were measured [39]. Each treatment was repeated three times.

### 2.11. Assay of B. mojavensis B1302 Culture Filtrate on Disease Control

In potted plants experiments, antagonistic strain B1302 were examined for their biocontrol efficacies against wheat sharp eyespot. Triadimefon, carbendazim, *Validamycin*·*Bacillus*, and sterile water were used as positive and negative controls, respectively. Triadimefon, carbendazim, *Validamycin*·*Bacillus*, sterile water, and cultivation liquid of the B1302 bacterial strain suspension were sprayed separately onto the wheat. The potted plants experiments were repeated three times. After infection with the disease in the wheat in the control group, the incidence of wheat sharp eyespot was statistically analyzed according to a scale ranging from 0 to 9 (0 = 0%; 1 ≤ 5%; 3 = 6–15%; 5 = 16–25%; 7 = 26–50%; and 9 ≥ 50%). The disease index and control efficacy were calculated as follows [40]:

Disease index = ∑ ((Number of diseased stems in each class × Number of the severity classes) ÷ (Total number of stems investigated × The highest severity class)) × 100.

Control efficacy (%) = ((Disease index of control − Disease index of treatment group) ÷ Disease index of control) × 100.

### 2.12. Toxicological Evaluation in Mice

An optimal cultivation medium and conditions were used to prepare the culture filtrate. The culture filtrate was filtered, concentrated, dried, ground, and sterilized to make powder.

Twenty healthy KM mice were randomly divided into two groups, with ten males and ten females in each group. Animal rooms were maintained at a temperature of 20–22 °C with a relative humidity of 40–70%, 12 h light/dark cycle and air ventilation at 18 times per hour. All animal studies protocols were approved by the Ethical Committee of Henan University of Technology, and all operations accorded with the guidelines and regulations of Henan University of Technology.

A maximum tolerated dose (MTD) study was carried out on healthy KM mice according to the method of Lu et al. [41]. The KM mice were orally administered with the initial 0.2 mL of 50 mL/kg culture filtrate powder and with the same amount of sterile water as the control group. The weights of the mice in each group were weighed on day 0, 2, 4, 8, and 14, respectively; the poisoning performance and death of the mice were observed; the changes in the fur, eyes, limb activities, and behavior of the mice were recorded; and symptoms of convulsion, diarrhea, salivation, lethargy, and coma were noted [42].

Blood assays were performed to determine the glutamic-pyruvic transaminase, glutamic-oxalacetic transaminease, total bilirubin, urea nitrogen, and serum creatinine levels by biochemical analyzer. Additionally, the KM mice were dissected, and organs (heart, liver, spleen, kidneys, and lungs) were collected and examined. The relative organ weight of each organ was then calculated [43].

The relative organ weight (%) = (Weight of organ ÷ Total weight of KM mice) × 100

All tests were repeated three times.

### 2.13. Statistical Analysis

A statistical analysis of the data was performed using the Sigma statistical software (SPSS 22.0, IBM, Chicago, IL, USA). The results were expressed as the mean ± SE. The statistical significance was determined by a one-way analysis of variance (ANOVA) and the post hoc test–least square difference (LSD) test. Differences were considered significant at *p* < 0.05.

## 3. Results

### 3.1. Isolation and Screening of Antagonistic Bacteria against R. cerealis

**Figure 1:**
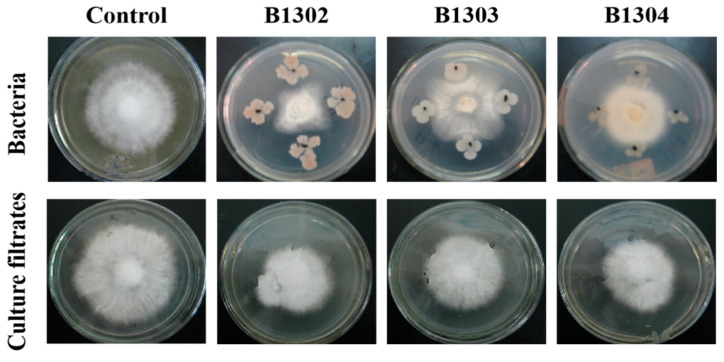
Inhibitory effects of bacteria B1302 and its culture filtrate on *R. cerealis* on PDA.

Twenty-five isolates were obtained from the rhizosphere soil of wheat. Among them, only three isolates showed inhibitory effects on *R. cerealis* in the confrontation plate (Figure 1). Strain B1302, which had been isolated from the soil, showed the highest inhibitory activity against *R. cerealis*.

### 3.2. Identification of Antagonistic Strain B1302

Morphological observations revealed that antagonistic bacteria B1302 was light yellow-brown with white, opaque colony edges. The bacteria produce acids, not gases, from glucose decomposition and galactose decomposition. The gram-staining, catalase test, citrate utilization, methyl-red test, Voges–Proskauer test, and starch hydrolysis test were all positive (Table 3).

**Figure 2:**
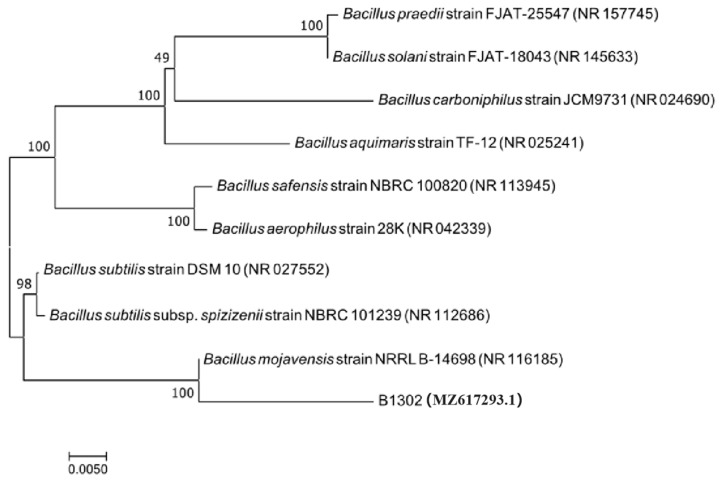
Phylogenetic tree based on the 16S rDNA sequence of B1302 and their homologous sequences.

To identify B1302, sequences of the 16S rDNA was obtained and analyzed through BLAST against the GenBank database. Based on the 16S rDNA sequence alignments, a phylogenetic tree was constructed (Figure 2). Strain B1302 showed the highest homology (100%) with *B. mojavensis* NRRL B-14698 (NR 116285), and these strains were clustered on the same evolutionary branch as *B. mojavensis.*

### 3.3. Broad Antifungal Activity of B. mojavensis B1302

**Figure 3:**
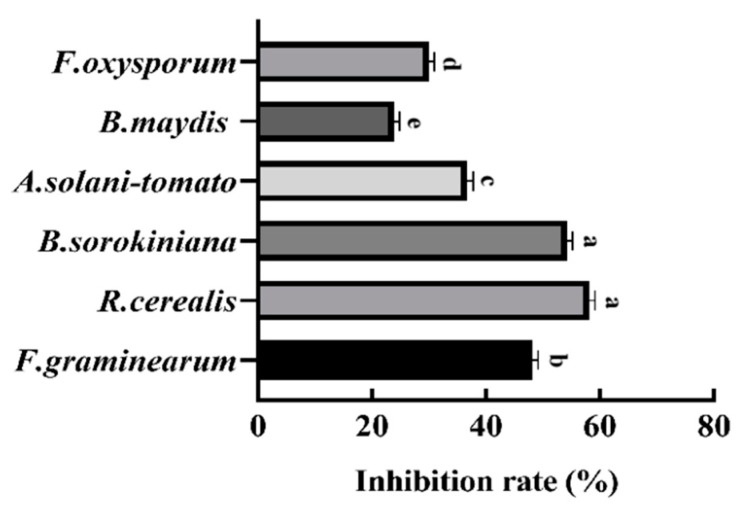
Antifungal activity of the B1302 strain against six pathogens. Bars labelled with different letters are significantly different at *p*  <  0.05 using a Duncan’s multiple range test.

*B. mojavensis* B1302 had antifungal activity toward six plant pathogens (Figure 3). The inhibitory activity of B1302 against *R. cerealis* was the highest inhibition rate (59.24%), and it was weakest against *B. maydis* (23.86% inhibition). The results showed that strain B1302 had obvious inhibitory activity against the pathogens.

### 3.4. Optimal Culture Conditions

**Figure 4:**
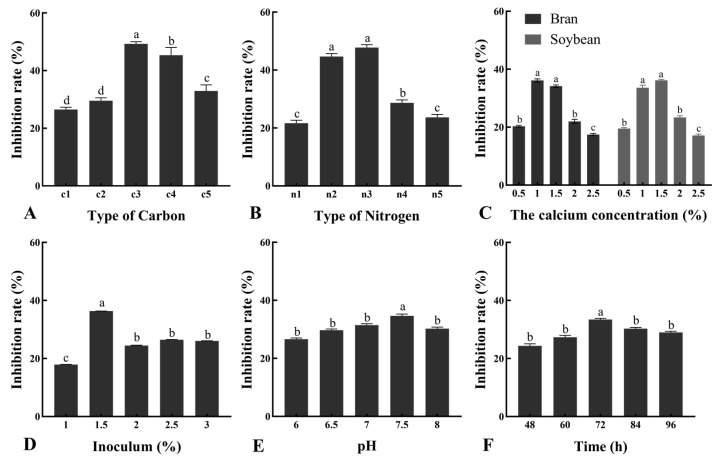
Effect of cultivation medium components and cultural conditions on the activities of strain B1302 against *R. cerealis*. (**A**), Carbon (c1, corn; c2, glycerin; c3, wheat bran; c4, sucrose; c5, glucose); (**B**), Nitrogen (n1, peptone; n2, yeast extract; n3, soybean meal; n4, soybean powder; n5, NaNO_3_); (**C**), Calcium concentration; (**D**), Inoculation amount of bacteria; (**E**), pH; (**F**), cultivation time. Data in the figure are the mean ± SE. Different lower-case letters on the bars indicate the significant difference at the *p* < 0.05 level using Duncan’s multiple range test.

Cultural conditions have a significant effect on the inhibition activity of strain B1302 against *R. cerealis* (Figure 4). The optimal culture conditions of antagonistic bacteria B1302 were determined by response surface methodology. The best culture conditions were as follows: the concentration of carbon source (wheat bran) is 10 g/L, the concentration of nitrogen source (soybean meal) is 15 g/L, rotation speed is 224.23 r/min, initial pH is 7.50, inoculation amount is 1.5 × 10^8^ CFU/mL, temperature is 35 °C, and cultivation time is 72 h. Under these conditions, the inhibition rate was 69.26%. The conditions described above can be used as the most effective culture conditions for strain B1302 to control wheat sharp eyespot.

### 3.5. Optimization of Media Composition

#### 3.5.1. Screening of Cultivation Parameters

The screening of cultivation parameters was conducted by employing a Plackett–Burman design of experiments to determine their influence on the inhibition rate of B1302 cultivation broth. The results were shown in Table 4. According to Table 4, influences from 7 factors on the inhibition rate were X5 > X6 > X2 > X1 > X3 > X7 > X4. Furthermore, X5, X6, X2, and X1 had significant influences on the test results and were taken as the main effect factors. However, according to the benefit and value of biological control, the 3 main factors were X5 (speed), X2 (soybean meal concentration), and X1 (wheat bran concentration).

#### 3.5.2. Optimization of Cultivation Conditions

Based on the results of the PB test, the cultivation conditions of antagonistic bacteria B1302 were carried out in three factors for three levels with the Box–Benhnken design by Design-Expert software 8.0.6 (Stat-Ease, Inc., Minneapolis, MN, USA). Each test was repeated 3 times and the results were shown in Table 5. The variance analysis is shown in Table 6.

The regression equation for the multiple regression fitting was used to analyze the response value by Design-Expert software 8.0.6 and the regression equation of the inhibition rate was obtained.

It was shown in Table 6 that the regression model F = 302.12, *p* < 0.0001 and the regression equation were very significant. The *p*-value of the Unintended term was 0.0920 > 0.05 and was not significant, which indicates that the error of the test is small, and the unknown factors have less interference on the results. The determination coefficient of the model was *R*^2^ = 0.9974, indicating better model fitting. Adjusted *R*^2^*adj* = 0.9941 showed that variation in the regression equation can explain the inhibitory rate of the results at a degree of 99.41%. The coefficient of variation (CV) is 2.40% less than 10%, which indicates that the model can better respond to the true value of the test.

The coefficient *p*-value of the model was analyzed. The effect of factor A was not significant (*p* > 0.05), and other factors had a very significant influence on the inhibition rate. The order of the three factors was rotational speed (C) > concentration of soybean meal (B) > concentration of wheat bran (A).

**Figure 5:**
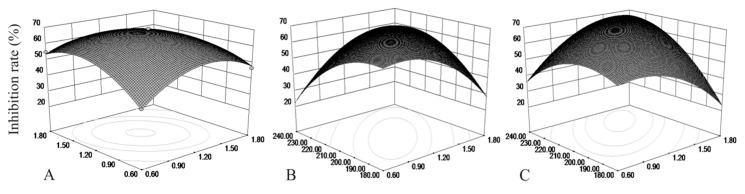
Response surface plots and contour lines of the effects of interaction between the concentrations of bran, soybean meal, and rotation speed on inhibition rate. (**A**) Wheat bran/%, (**B**) Soybean meal/%, (**C**) Speed/(r·min^−1^)).

The interaction curves and the contour map of the concentration of bran, soybean meal, and rotation speed on the inhibition rate were obtained by Design-Expert software 8.0.6. The results are shown in Figure 5.

The response surface can directly reflect the influence of each factor on the response value. The steepening of the surface indicates the stronger interaction between the related factors. From Figure 5, we can see that the interactions between the concentrations of wheat bran, soybean meal, and rotation speed have an elliptical effect on the inhibition rate. It indicated that the interaction is more significant. The response surface line corresponding to the rotational speed is steeper, which indicates that the speed has a significant influence on the inhibition rate; this is in accordance with the variance analysis of the regression equation.

To verify the accuracy and reliability of the optimized culture conditions, the culture conditions were adjusted to 13.2 g/L of wheat bran, 14.1 g/L of soybean meal, and a 224 r/min rotation speed. The other conditions remain unchanged, and the test was repeated three times. The final inhibition rate was 67.44% and had a relative error that was 2.6% less than the theoretical value. This model further showed that the prediction value fits well with the actual value, and that the optimized culture conditions of antagonistic bacteria B1302 is accurate and feasible.

### 3.6. Antagonistic Activity of B1302 Culture Filtrates

**Figure 6:**
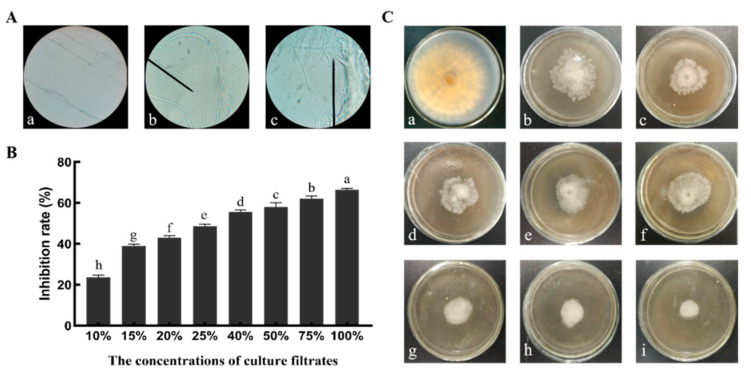
Effect of the B1302 culture filtrate treatment on the growth of *R. cerealis* mycelia. (**A**), mycelia morphological characteristics of *R. cerealis* cultivated on PDA medium ((**a**), control; (**b**), broken; (**c**), shriveled). (**B**), Inhibition rate of different concentrations of the B1302 culture filtrates on mycelial growth rate; Different lower-case letters on the bars indicate the significant difference at the *p* < 0.05 level using Duncan’s multiple range test. (**C**), effect of different concentrations of the B1302 culture filtrates on mycelial growth rate in Petri dishes ((**a**), 0%; (**b**), 10%; (**c**), 15%; (**d**), 20%; (**e**), 25%; (**f**), 40%; (**g**), 50%; (**h**), 75%; (**i**), 100%) on mycelial growth rate.

The inhibitory effects of B1302 culture filtrates on the growth of *R. cerealis* were investigated by plate tests (Figure 6C). In vitro experiments showed that B1302 culture filtrates with concentrations between 10–100% significantly inhibited the growth of *R. cerealis* and exhibited a dose-dependent effect (Figure 6B).

The light microscopy results showed that the hyphae were healthy, uniform, and linear, and did not show any abnormalities in the control, whereas hyphae treated by B1302 culture filtrates were abnormal, broken, and shriveled in appearance (Figure. 6A).

### 3.7. Extracellular Enzyme Activities and Detection of PGP Abilities

In the assays for the secreted extracellular enzymes, bacterial strain B1302 formed hydrolytic circles on the plate. The results showed that strain B1302 could produce β-1,3 glucanase, protease, and chitinase.

**Figure 7:**
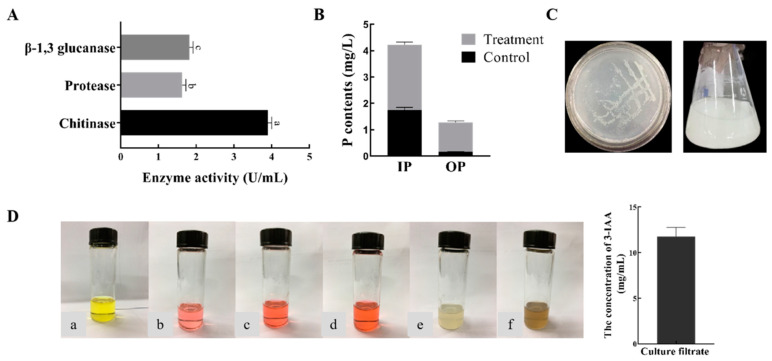
Extracellular enzyme activities and assays for detection of PGP abilities. (**A**), Extracellular enzyme activities (β-1,3 glucanase, protease, chitinase); Different lower-case letters on the bars indicate the significant difference at the *p* < 0.05 level using Duncan’s multiple range test. (**B**), Solubilize phosphate (Inorganic phosphorous (IP); Organic phosphorus (OP)); (**C**), Nitrogen-fixation plate and shake flask test; (**D**), IAA production and content (Colorimetric analysis was conducted on compounds derived from Indole-3-Acetic Acid (IAA). ((**a**), colorimetric solution + sterile water; (**b**), colorimetric solution + 10 mg/L IAA; (**c**), colorimetric solution + 30 mg/L IAA; (**d**), colorimetric solution + 50 mg/L IAA; (**e**), colorimetric solution + LB borth; (**f**), colorimetric solution + sterile filtrate).

Plant growth-promoting (PGP) traits such as phosphate solubilization, IAA biosynthesis, and nitrogen-fixation were shown in Figure 7. B1302 was able to solubilize phosphate and produce IAA, even without any L-tryptophan induction. The strain was also able to grow in a nitrogen-free medium, indicating its ability to fix atmospheric nitrogen.

### 3.8. Wheat Growth Promotion by B. mojavensis B1302 Culture Filtrate

The effects of different treatments on seed germination rate, height, and fresh weight of wheat are shown in Table 7. The results show that the pesticide (Triadimefon, Carbendazim, and *Validamycin*·*Bacillus*) treatment group and antagonistic bacteria B1302 culture filtrate promoted the growth of wheat seedlings and reached a very significant level compared to the control.

### 3.9. Effects of B. mojavensis B1302 Culture Filtrate on Disease Control

In the potted plants experiments, the results showed that B1302 culture filtrate, triadimefon, carbendazim, and *Validamycin*·*Bacillus* treatment showed a higher control efficacy (>61%) on wheat sharp eyespot than that in the control (Table 8). In addition, differences between *B. mojavensis* B1302 culture filtrate and commercially available fungicides were not significant. In conclusion, the treatment of strain *B. mojavensis* B1302 significantly reduced disease severity and showed better control on wheat sharp eyespot.

### 3.10. Safty Evaluation of B1302 Culture Filtrate on Mice

After the oral administration of *B. mojavensis* B1302 culture filtrate powder to mice and observing their actions for 14 days, there were no deaths in the control group and experimental group. No changes in other physiological activities were observed immediately after administration of *B. mojavensis* B1302 culture filtrate powder or during the post-treatment period in the two groups. As shown in Table 9, the final weight was one of the few differences between the experimental group and control group.

According to Table 10, after intragastric administration of the culture filtrate of *B. Mojave* B1302 to mice for 14 days, mouse organs were dissected. The organs indicate that the mice, including the heart, liver, and spleen, did not differ significantly between the control and treatment groups (*p* > 0.05).

The blood of the two mice groups was collected for major organ toxicity assessment. The levels of serum AST, ALT, CREA, and BUN showed no significant differences (Table 11). The results indicated that this dose caused no damage to mice and that the B1302 culture filtrate was safe for animals.

## 4. Discussion

Biological control agents can prevent plants from pathogens. Among the many advantages of biological control agents is their environmental-friendliness [44]. Common root rot (*Fusarium*) and wheat sharp eyespot (*R. cerealis*) are economically important diseases in global wheat [45]. Isolated microbes from the environment, such as soils or plants, are often the first step toward biological effects. In the present study, we screened 25 strains for antagonistic effects against bacteria from soil samples, and 1 of these showed high antagonism against *R. cerealis*. Then, the biochemical, physiological, and phylogenetic analyses of the 16S rDNA gene sequences confirmed that strain B1302 was *B. mojavensis*. During the biocontrol of plant diseases, the screening of single resistant strains still mostly dominates. Hena Jamali et al., isolated *Bacillus subtilis* RH5 from saline soil, which exhibited antifungal activity to the fungal pathogen, *R. solani* [46]. Zhao et al., isolated *Bacillus subtilis* strain SG6 from wheat kernels and plant anthers against *F. graminearum* [47]. In the present study, by using confronting incubation, strain B1302 exhibits different degrees of inhibition for *R. cerealis, F. Graminearum, B. sorokiniana; A. solani, B. maydis Shoem,* and *F. oxysporum.* Among them, it exhibited stronger antifungal activity against *R. cerealis* and showed broad-spectrum antifungal activity.

Nevertheless, medium composition strongly influenced microbial growth and the accumulation of metabolic products, and optimizing these parameters can improve bacterial efficiency. The single-factor method and response surface methodology (RSM) were the optimization techniques employed. Different carbon sources were used as carbon sources for antimicrobial substance production. Our results indicated that wheat bran fulfills the requirement of a carbon source and acts as the most significant substrate. Moreover, the type of nitrogen source is as equally important as the nutritional requirements of the organism. In this study, soybean meal was found to be the most effective for antifungal activity. Coronel-León et al., reported that the maximum antimicrobial substance of *B. licheniformis* AL1.1 was obtained using 1.5% glucose [48]. We also found that the ideal concentration of wheat bran is 13.2 g/L and the concentration of soybean meal is 14.1 g/L, which can reach better antifungal activity. Moreover, the inoculation volume directly affects the microbial cultivation efficiency. Antifungal activity will be most effective when the inoculation amount of bacteria reached 1.5 × 10^8^ CFU/mL. Finally, the results proved that cultivation pH and time also affected antimicrobial substance production. A pH of 7.50 and cultivation time of 72 h, which had the best efficiency, is ideal. Based on the above results, we further optimized the cultivation medium formula with an orthogonal test. In the present study, the significant variables necessary for the enhancement of antimicrobial substance production were selected using the Plackett–Burman design. The RSM applied to the optimization of antimicrobial substance production in this investigation suggested the importance of a variety of factors at different levels. Overall, it can be concluded that optimization medium was more favorable for significant bacterial growth.

A study of the antifungal ability and the change of mycelial morphology of the pathogen was carried out in the present study. It is generally known that inhibition of hyphal growth is the main pattern of *Bacillus* spp. strains against *R. cerealis*. Yang et al., reported that *Bacillus subtilis* strain YB-05 changed the mycelium of pathogen GGT-007, which were characterized by enlarged hyphae, abnormal shape, vesicles, distortion, or empty cells devoid of cytoplasm [49]. Saoussen Ben Khedher et al., also indicated that *Bacillus subtilis* V26 also led to the swelling and deformation of *Botrytis cinerea* hyphae [50]. The result showed that B1302 culture filtrates significantly exhibit inhibition for *R. cerealis* and a concentration-dependent ability in vitro. Through light microscopy, we also found that B1302 culture filtrates can lead to abnormal hyphae growth, as well as hyphae that appear broken and shriveled in appearance.

The report indicates that *Bacillus* spp. strains differ in their capacity to produce and secrete bioactive metabolites, even if they belong to the same species [51]. For the mode of action of *Bacillus* spp. with pathogenic bacteria, it is usually the ability to produce some extracellular enzymes (such as protease, chitinase, β-1,3-glucanase, and cellulase) that destroys the cellular structure of the pathogenic bacteria. Nevertheless, *Bacillus* spp. still have the ability to produce PGP activity to promote crop growth. Given these outstanding abilities, researchers have progressively studied the genus *Bacillus.* Zhou et al., reported that *Bacillus cereus* YN917 had produced IAA, ACC deaminase, siderophores, protease, amylase, cellulase, and β-1,3-glucanase, and harbored mineral phosphate decomposition activity [52]. Ei Mon Myo et al., proved that *Bacillus velezensis* NKG-2 produced chitinase, cellulase, β-glucanase, IAA, and siderophore [53]. It is reported that different genera have different enzyme production capacities. In the present study, *B. mojavensis* strain B1302 produced extracellular enzyme activity and various PGP substances, such as, β-1,3 glucanase, protease, chitinase, and IAA, and has nitrogen-fixation, phosphorus-solubilizing activity, which helps plants to survive under *R. cerealis* infection. Similarly, the production of IAA and nitrogen-fixation, phosphorus-solubilizing activities enhance nutrient contents in plants, thereby leading to better growth and resistance under stress.

A potted plants experiment was performed to evaluate the effects of *B. mojavensis* B1302 on wheat. This study previously revealed the capability of this strain to produce extracellular enzymes and detect PGP abilities. Therefore, this prompted us to further investigate the efficiency in vivo. In this study, wheat seeds treated with B1302 culture filtrate had higher germination rates than those in the control. The reason may be due to the synthesis of hormones such as IAA, which can trigger the activity of specific enzymes that promote early germination. In the potted plants experiment, the increased wheat height and fresh weight implied that the B1302 strain could play a role in promoting growth. Also, *B. mojavensis* B1302 strain exhibits a very good control efficacy compared to commercial fungicides. Then, the strain was tested for its impact on disease resistance of the pathogens in a pot trial. *B. mojavensis* B1302 culture filtrate could reduce the disease index. In addition, we examined the efficacies of culture filtrate and commercial fungicide. B1302 culture filtrate had a higher efficacy (65.25%) than that in the control group, and it showed that culture filtrate treatment effects did not vary significantly with the commercial fungicide. Therefore, *B. mojavensis* B1302 had better control effects against *R. cerealis* and is consistent with the antifungal effects in vitro.

However, since the microorganisms investigated in this research are from a natural source, they are anticipated to be harmless to humans. Given this, in the next step, we also carried out animal experiments to validate our findings. After two weeks of feeding and gavage with B1302 culture filtrate powder, all mice were alive and healthy. Furthermore, we also found that there was no distinct difference between the two groups in the organ index and serum biochemical index. Therefore, culture filtrate of *B. mojaves* B1302 is safe, and is consistent reports that the antagonistic strain and its cultivation products are non-toxic [54].

## 5. Conclusions

Antagonistic strain B1302 was isolated and identified as *B. mojavensis* B1302. Culture filtrate of strain B1302 had a broad antifungal spectrum. The final optimized medium composition and culture conditions were 13.2 g/L of wheat bran, 14.1 g/L of soybean meal, a rotation of speed of 224 r/min, 7.50 initial pH, and 1.5 × 10^8^ CFU/mL of inoculation amount at 35°C for a 72-h culture duration. *B. mojavensis* B1302 inhibited the hyphae growth of *R. cerealis* and produced hydrolysis enzymes (protease, chitinase, and glucanase) and IAA, and had N-fixing potentiality and P-solubilisation capacity. It can also promote wheat seedling growth and had a control efficacy of 65.25%, which is consistent with the effects of commercial pesticides. An animal toxicological assay showed that the culture filtrate is safe and has the potential to be developed into microbial fungicides.

## Figures and Tables

**Table 1:** Supplements for the screening of factors using Plackett–Burman method.

**Number**	**Factors**	**−1**	**1**
X1	Concentration of wheat bran/%	0.5	2.0
X2	Concentration of soybean meal/%	0.6	2.0
X3	pH	6.0	8.0
X4	Temperature/°C	30.0	40.0
X5	Speed/(r/min)	180.0	240.0
X6	Inoculation/%	1.0	3.0
X7	cultivation time/h	48.0	72.0

**Table 2:** Variables and levels for Box–Behnken design for the optimization of parameters.

**Factors**	**Code**	**−1**	**0**	**1**
Concentration of wheat bran/%	A	0.6	1.2	1.8
Concentration of soybean meal/%	B	0.6	1.2	1.8
Speed/(r/min)	C	180	210	240

**Table 3:** Physiological and biochemical characteristics of the isolated bacterial strains.

Characteristics	B1302	B1303	B1304
Gram staining	+	+	-
Colony color (visual test)	Yellowish-brown	Lactic	Light yellow
Glucose decomposition	Acids, no gas	Acids, no gas	Acids, no gas
Galactose decomposition	Acids, no gas	Acids, no gas	Acids, no gas
Catalase test	+	+	+
Citrate utilization	+	-	+
Methyl-Red test	+	+	+
Phenylalanine deaminase test	-	-	-
Voges–Proskauer test	+	+	+
Starch hydrolysis test	+	-	-

**Table 4:** Plackett–Burman experimental design and response value.

Numbers	X1	X2	X3	X4	X5	X6	X7	Y (Inhibition Rate)/%
1	1	1	1	−1	1	−1	−1	26.84
2	1	−1	−1	1	1	1	−1	27.59
3	−1	1	1	1	−1	1	−1	22.28
4	1	1	−1	−1	−1	1	1	16.35
5	−1	1	1	−1	−1	−1	1	23.49
6	1	−1	−1	−1	1	−1	1	27.54
7	−1	1	−1	−1	1	1	−1	34.56
8	−1	−1	1	−1	1	1	1	40.87
9	1	1	1	1	1	1	1	29.27
10	−1	−1	1	1	1	−1	−1	31.71
11	1	−1	1	−1	−1	1	−1	28.10
12	−1	−1	−1	1	−1	1	1	37.43
13	1	−1	1	1	−1	−1	1	21.93
14	1	1	−1	1	−1	−1	−1	11.05
15	−1	1	−1	1	1	−1	1	23.63
16	−1	−1	−1	−1	−1	−1	−1	20.95

**Table 5:** Box–Benhnken experimental design and response value.

Numbers	A	B	C	Y (Inhibition Rate)/%
1	1	−1	0	46.68
2	−1	1	0	54.91
3	0	0	0	68.26
4	1	0	1	63.51
5	1	1	0	39.56
6	0	0	0	67.94
7	0	1	−1	29.27
8	−1	0	−1	59.34
9	0	1	1	64.61
10	0	−1	1	32.39
11	0	−1	−1	56.17
12	−1	−1	0	38.92
13	0	0	0	68.10
14	−1	0	1	29.70
15	0	0	0	69.85
16	1	0	−1	24.84
17	0	0	0	68.10

**Table 6:** Variance analysis of regression equation.

Type	Sum of Squares	Degree of Freedom	Average of Squares	*F* Value	*p* Value	Significance
Model	4206.01	9	467.33	302.12	<0.0001	**
*A*	8.57	1	8.57	5.54	0.0508	
*B*	25.17	1	25.17	16.27	0.0050	**
*C*	52.99	1	52.99	34.26	0.0006	**
*AB*	133.52	1	133.52	86.32	<0.0001	**
*AC*	1166.26	1	1166.56	754.16	<0.0001	**
*BC*	873.79	1	873.79	564.89	<0.0001	**
*A^2^*	641.94	1	641.94	415.00	<0.0001	**
*B^2^*	517.38	1	517.38	334.47	<0.0001	**
*C^2^*	581.81	1	581.81	376.13	<0.0001	**
Residual	10.83	7	1.55			
Unintended term	8.33	3	2.78	4.44	0.0920	
Pure error	2.50	4	0.63			
Total error	4216.84	16				

‘**’ indicated extremely significant difference (*p* < 0.01).

**Table 7:** Promotion of different treatments on the growth of wheat seedlings.

Treatments	Germination Rate (%)	Height/mm	Fresh Weight/g
Control	81 ± 4.05	82.26 ± 4.11	0.67 ± 0.034
*B. mojavensis* B1302	90 ± 4.50	102.15 ± 5.11	1.02 ± 0.051
Triadimefon	99 ± 4.95	108.35 ± 5.42	1.02 ± 0.051
Carbendazim	85 ± 4.25	104.36 ± 5.22	1.034 ± 0.05
*Validamycin·Bacillus*	89 ± 4.45	89.86 ± 4.49	0.68 ± 0.033

**Table 8:** Biocontrol efficacy of different treatments on wheat sharp eyespot.

Treatments	Disease Incidence Rate (%)	Disease Index	Control Efficacy (%)
Control	95.36 ± 3.12	81.47 ± 3.31	--
*B. mojavensis* B1302	59.42 ± 3.06	28.31 ± 1.63	65.25
Triadimefon	53.14 ± 4.27	25.83 ± 3.29	68.29
Carbendazim	60.38 ± 1.93	31.18 ± 2.57	61.73
*Validamycin*·*Bacillus*	48.32 ± 3.06	23.63 ± 3.17	70.99

**Table 9:** Effects of B1302 culture filtrate on the weight of mice in each group.

Items		Days (d)				
		0 d	2 d	4 d	8 d	14 d
Weight	Control group ♂	19.84 ± 1.74 ^a^	22.05 ± 1.80 ^a^	24.02 ± 1.87 ^a^	27.79 ± 1.87 ^a^	35.79 ± 1.83 ^a^
	Treatment group ♂	19.64 ± 1.78 ^a^	21.70 ± 1.01 ^a^	23.36 ± 1.24 ^a^	28.09 ± 1.76 ^a^	36.76 ± 1.51 ^a^
	Control group ♀	19.23 ± 1.67 ^a^	20.58 ± 1.54 ^a^	23.18 ± 1.49 ^a^	26.76 ± 1.81 ^a^	32.86 ± 1.01 ^a^
	Treatment group ♀	19.16 ± 1.80 ^a^	20.30 ± 1.65 ^a^	22.54 ± 1.63 ^a^	26.28 ± 1.88 ^a^	30.19 ± 1.72 ^a^

“♂” represents male mice; “♀” represents female mice; ^a^ Different lower-case letters on the right shoulder indicate the significant difference at the *p* < 0.05 level using Duncan’s multiple range test; The same letter represents no significant differences between treatments.

**Table 10:** Effects of B1302 culture filtrate on organ index of mice in each group.

Items	Organ Index (%)				
	Heart	Lung	Liver	Spleen	Kidney
Control group ♂	0.56 ± 0.0011 ^a^	0.63 ± 0.0007 ^a^	4.19 ± 0.0054 ^a^	0.19 ± 0.0002 ^a^	1.56 ± 0.0022 ^a^
Treatment group ♂	0.52 ± 0.0005 ^a^	0.76 ± 0.0010 ^a^	4.27 ± 0.0036 ^a^	0.21 ± 0.0004 ^a^	1.70 ± 0.0014 ^a^
Control group ♀	0.58 ± 0.0008 ^a^	0.76 ± 0.0012 ^a^	5.08 ± 0.0109 ^a^	0.23 ± 0.0007 ^a^	1.41 ± 0.0017 ^a^
Treatment group ♀	0.56 ± 0.0008 ^a^	0.77 ± 0.0005 ^a^	5.06 ± 0.0039 ^a^	0.23 ± 0.0007 ^a^	1.24 ± 0.0018 ^a^

“♂” represents male mice; “♀” represents female mice; ^a^ Different lower-case letters on the right shoulder indicate the significant difference at the *p* < 0.05 level using Duncan’s multiple range test.; The same letter represents no significant differences between treatments.

**Table 11:** The serological physiological indexes of mice in each group.

Items	Serological Physiological Index				
	ALT (U·L^−1^)	AST (U·L^−1^)	ALT/AST (U·L^−1^)	BUN (mmoL·L^−1^)	CREA (μmol·L^−1^)
Control group ♂	90.37 ± 9.23 ^a^	161.53 ± 17.42 ^a^	1.78 ± 0.26 ^a^	12.20 ± 2.43 ^a^	17.30 ± 3.01 ^a^
Treatment group ♂	89.43 ± 13.47 ^a^	163.60 ± 19.19 ^a^	1.82 ± 0.55 ^a^	12.90 ± 1.18 ^a^	17.25 ± 3.74 ^a^
Control group ♀	41.94 ± 2.01 ^a^	120.24 ± 9.90 ^a^	2.86 ± 0.44 ^a^	13.50 ± 2.02 ^a^	20.44 ± 1.63 ^a^
Treatment group ♀	41.48 ± 5.12 ^a^	122.08 ± 12.57 ^a^	2.94 ± 0.50 ^a^	12.85 ± 2.83 ^a^	20.43 ± 2.32 ^a^

“♂” represents male mice; “♀” represents female mice; ^a^ Different lower-case letters on the right shoulder indicate the significant difference at the *p* < 0.05 level using Duncan’s multiple range test.; The same letter represents no significant differences between treatments.

## Data Availability

Not applicable.

## References

[1] Francesconi S., Harfouche A., Maesano M., Balestra G.M. (2021). UAV-Based Thermal, RGB Imaging and Gene Expression Analysis Allowed Detection of *Fusarium* Head Blight and Gave New Insights into the Physiological Responses to the Disease in Durum Wheat. Front. Plant Sci..

[2] Schneider J., Berkner M.O., Philipp N., Schulthess A.W., Reif J.C. (2021). Assessing the Suitability of Elite Lines for Hybrid Seed Production and as Testers in Wide Crosses with Wheat Genetic Resources. Front. Plant Sci..

[3] Fan X., Xu Z., Wang F., Feng B., Zhou Q., Cao J., Ji G., Yu Q., Liu X., Liao S. (2020). Identification of colored wheat genotypes with suitable quality and yield traits in response to low nitrogen input. PLoS ONE.

[4] Lemańczyk G., Kwaśna H. (2013). Effects of sharp eyespot (*Rhizoctonia cerealis*) on yield and grain quality of winter wheat. Eur. J. Plant Pathol..

[5] Shin J., Park B., Kim H., Lee K., Kim K.S. (2021). Antagonistic and Plant Growth-Promoting Effects of *Bacillus velezensis* BS1 Isolated from Rhizosphere Soil in a Pepper Field. Plant Pathol. J..

[6] Hamada M.S., Yin Y., Chen H., Ma Z. (2011). The escalating threat of *Rhizoctonia cerealis*, the causal agent of sharp eyespot in wheat. Pest Manag. Sci..

[7] Wang M., Zhu X., Wang K., Lu C., Luo M., Shan T., Zhang Z. (2018). A wheat caffeic acid 3-O-methyltransferase Ta-COMT-3D positively contributes to both resistance to sharp eyespot disease and stem mechanical strength. Sci. Rep..

[8] Zhao X., Song P., Hou D., Li Z., Hu Z. (2021). Antifungal activity, identification, and biosynthetic potential analysis of fungi against *Rhizoctonia cerealis*. Ann. Microbiol..

[9] Li W., Hu M., Xue Y., Li Z., Zhang Y., Zheng D., Lu G., Wang J., Zhou J. (2020). Five Fungal Pathogens Are Responsible for Bayberry Twig Blight and Fungicides Were Screened for Disease Control. Microorganisms.

[10] Cao Y., Pi H., Chandrangsu P., Li Y., Wang Y., Zhou H., Xiong H., Helmann J.D., Cai Y. (2018). Antagonism of Two Plant-Growth Promoting *Bacillus velezensis* Isolates Against *Ralstonia solanacearum* and *Fusarium oxysporum*. Sci. Rep..

[11] Lohse R., Jakobs-Schonwandt D., Patel A.V. (2014). Screening of liquid media and fermentation of an endophytic *Beauveria bassiana* strain in a bioreactor. AMB Express.

[12] Besset-Manzoni Y., Joly P., Brutel A., Gerin F., Soudiere O., Langin T., Prigent-Combaret C. (2019). Does in vitro selection of biocontrol agents guarantee success *in planta*? A study case of wheat protection against *Fusarium* seedling blight by soil bacteria. PLoS ONE.

[13] Chaiharn M., Theantana T., Pathom-Aree W. (2020). Evaluation of Biocontrol Activities of *Streptomyces* spp. against Rice Blast Disease Fungi. Pathogens.

[14] Li B., Li Q., Xu Z., Zhang N., Shen Q., Zhang R. (2014). Responses of beneficial *Bacillus amyloliquefaciens* SQR9 to different soilborne fungal pathogens through the alteration of antifungal compounds production. Front. Microbiol..

[15] Krishnamoorthy R., Kwon S.W., Kumutha K., Senthilkumar M., Ahmed S., Sa T., Anandham R. (2018). Diversity of culturable methylotrophic bacteria in different genotypes of groundnut and their potential for plant growth promotion. 3 Biotech.

[16] Kim M.J., Shim C.K., Park J. (2021). Control Efficacy of *Bacillus velezensis* AFB2-2 against Potato Late Blight Caused by *Phytophthora infestans* in Organic Potato Cultivation. Plant Pathol. J..

[17] Li Y., Heloir M.C., Zhang X., Geissler M., Trouvelot S., Jacquens L., Henkel M., Su X., Fang X., Wang Q. (2019). Surfactin and fengycin contribute to the protection of a *Bacillus subtilis* strain against grape downy mildew by both direct effect and defence stimulation. Mol. Plant Pathol..

[18] Chowdhury S.P., Uhl J., Grosch R., Alquéres S., Pittroff S., Dietel K., Schmitt-Kopplin P., Borriss R., Hartmann A. (2015). Cyclic lipopeptides of *Bacillus amyloliquefaciens* subsp. plantarum colonizing the lettuce rhizosphere enhance plant defense responses toward the bottom rot pathogen *Rhizoctonia solani*. Mol. Plant-Microbe Interact..

[19] Chen M., Wang J., Liu B., Zhu Y., Xiao R., Yang W., Ge C., Chen Z. (2020). Biocontrol of tomato bacterial wilt by the new strain *Bacillus velezensis* FJAT-46737 and its lipopeptides. BMC Microbiol..

[20] Ragul K., Syiem I., Sundar K., Shetty P.H. (2017). Characterization of probiotic potential of *Bacillus* species isolated from a traditional brine pickle. J. Food Sci. Technol..

[21] Rath M., Mitchell T.R., Gold S.E. (2018). Volatiles produced by *Bacillus mojavensis* RRC101 act as plant growth modulators and are strongly culture-dependent. Microbiol. Res..

[22] Bacon C.W., Hinton D.M. (2011). In planta reduction of maize seedling stalk lesions by the bacterial endophyte *Bacillus mojavensis*. Can. J. Microbiol..

[23] Blacutt A.A., Mitchell T.R., Bacon C.W., Gold S.E. (2016). *Bacillus mojavensis* RRC101 lipopeptides provoke physiological and metabolic changes during antagonism against *Fusarium verticillioides*. Mol. Plant-Microbe Interact..

[24] Ghazala I., Bouassida M., Krichen F., Manuel Benito J., Ellouz Chaabouni S., Haddar A. (2017). Anionic lipopeptides from *Bacillus mojavensis* I4 as effective antihypertensive agents: Production, characterization, and identification. Eng. Life Sci..

[25] Li S., Fang X., Zhang H., Zeng Y., Zhu T. (2019). Screening of Endophytic Antagonistic Bacterium from *Phellodendron amurense* and Their Biocontrol Effects against Canker Rot. Plant Pathol. J..

[26] Yi Y.J., Luan P.Y., Liu S.F., Shan Y.T., Hou Z.P., Zhao S.Y., Jia S., Li R.F. (2022). Efficacy of *Bacillus subtilis* XZ18-3 as a Biocontrol Agent against *Rhizoctonia cerealis* on Wheat. Agriculture.

[27] Islam M.R., Jeong Y.T., Ryu Y.J., Song C.H., Lee Y.S. (2009). Isolation, Identification and Optimal Culture Conditions of *Streptomyces albidoflavus* C247 Producing Antifungal Agents against *Rhizoctonia solani* AG2-2. Mycobiology.

[28] Zakry F.A.A., Shamsuddin Z.H., Rahim K.A., Zakaria Z.Z., Rahim A.A. (2012). Inoculation of *Bacillus sphaericus* UPMB-10 to young oil palm and measurement of its uptake of fixed nitrogen using the ^15^N isotope dilution technique. Microbes Environ..

[29] El-Helw N.O., El-Gendy A.O., El-Gebaly E., Hassan H.M., Rateb M.E., El-Nesr K.A. (2019). Characterization of natural bioactive compounds produced by isolated bacteria from compost of aromatic plants. J. Appl. Microbiol..

[30] Kumar S., Stecher G., Tamura K. (2016). MEGA7: Molecular evolutionary genetics analysis version 7.0 for bigger datasets. Mol. Biol. Evol..

[31] Plackett R.L., Burman J.P. (1946). The design of optimum multifactorial experiments. Biometrika.

[32] El-Naggar N.E., El-Shweihy N.M. (2020). Identification of cholesterol-assimilating actinomycetes strain and application of statistical modeling approaches for improvement of cholesterol oxidase production by *Streptomyces anulatus* strain NEAE-94. BMC Microbiol..

[33] Box G.E., Behnken D.W. (1960). Some new three level designs for the study of quantitative variables. Technometrics.

[34] Phoka N., Suwannarach N., Lumyong S., Ito S.I., Matsui K., Arikit S., Sunpapao A. (2020). Role of Volatiles from the Endophytic Fungus *Trichoderma asperelloides* PSU-P1 in Biocontrol Potential and in Promoting the Plant Growth of *Arabidopsis thaliana*. J. Fungi.

[35] Park J.H., Garcia C.V., Lee S.P. (2019). Fortification of Poly-**γ**-Glutamic Acid and **γ**-Aminobutyric Acid in Homogenized Hydroponic Ginseng Co-Fermented by *Bacillus subtilis* HA and *Lactobacillus plantarum* EJ2014. Prev. Nutr. Food Sci..

[36] Sen M., Sen S.P. (1965). Interspecific transformation in Azotobacter. J. Gen. Microbiol..

[37] Huang R., Chen P., Wang X., Li H., Zuo L., Zhang Y., Li L. (2020). Structural variability and niche differentiation of the rhizosphere and endosphere fungal microbiome of *Casuarina equisetifolia* at different ages. Braz. J. Microbiol..

[38] Szilagyi-Zecchin V.J., Ikeda A.C., Hungria M., Adamoski D., Kava-Cordeiro V., Glienke C., Galli-Terasawa L.V. (2014). Identification and characterization of endophytic bacteria from corn (*Zea mays* L.) roots with biotechnological potential in agriculture. AMB Express.

[39] Sharma S., Kulkarni J., Jha B. (2016). Halotolerant Rhizobacteria Promote Growth and Enhance Salinity Tolerance in Peanut. Front Microbiol..

[40] Gao X., Gong Y., Huo Y., Han Q., Kang Z., Huang L. (2015). Endophytic *Bacillus subtilis* strain E1R-J is a promising biocontrol agent for wheat powdery mildew. BioMed Res. Int..

[41] Lu L., Fan Y., Yao W., Xie W., Guo J., Yan Y., Yang F., Xu L. (2014). Safety assessment of the fermented *Phylloporia ribis* (*Lonicera japonica* Thunb.) mycelia by oral acute toxicity study in mice and 90-day feeding study in rats. Food Chem. Toxicol..

[42] Li P., Tian W., Jiang Z., Liang Z., Wu X., Du B. (2018). Genomic Characterization and Probiotic Potency of *Bacillus* sp. DU-106, a Highly Effective Producer of L-Lactic Acid Isolated from Fermented Yogurt. Front Microbiol..

[43] Algariri K., Atangwho I.J., Meng K.Y., Asmawi M.Z., Sadikun A., Murugaiyah V. (2014). Antihyperglycaemic and Toxicological Evaluations of Extract and Fractions of *Gynura procumbens* Leaves. Trop. Life Sci. Res..

[44] Chen P.H., Chen R.Y., Chou J.Y. (2018). Screening and Evaluation of Yeast Antagonists for Biological Control of *Botrytis cinerea* on Strawberry Fruits. Mycobiology.

[45] Guo F., Shan Z., Yu J., Xu G., Zhang Z. (2020). The Cysteine-Rich Repeat Protein TaCRR1 Participates in Defense against Both *Rhizoctonia cerealis* and *Bipolaris sorokiniana* in Wheat. Int. J. Mol. Sci..

[46] Sandvang D., Skjoet-Rasmussen L., Cantor M.D., Mathis G.F., Lumpkins B.S., Blanch A. (2021). Effects of feed supplementation with 3 different probiotic *Bacillus* strains and their combination on the performance of broiler chickens challenged with *Clostridium perfringens*. Poult. Sci..

[47] Jamali H., Sharma A., Roohi, Srivastava A.K. (2020). Biocontrol potential of *Bacillus subtilis* RH5 against sheath blight of rice caused by *Rhizoctonia solani*. J. Basic Microbiol..

[48] Coronel-León J., de Grau G., Grau-Campistany A., Farfan M., Rabanal F., Manresa A., Marqués A.M. (2015). Biosurfactant production by AL 1.1, a *Bacillus licheniformis* strain isolated from Antarctica: Production, chemical characterization and properties. Ann. Microbiol..

[49] Yang L., Quan X., Xue B., Goodwin P.H., Lu S., Wang J., Du W., Wu C. (2015). Isolation and identification of *Bacillus subtilis* strain YB-05 and its antifungal substances showing antagonism against *Gaeumannomyces graminis* var. tritici. Biol. Control..

[50] Ben Khedher S., Boukedi H., Laarif A., Tounsi S. (2020). Biosurfactant produced by *Bacillus subtilis* V26: A potential biological control approach for sustainable agriculture development. Org. Agric..

[51] Sun G., Yao T., Feng C., Chen L., Li J., Wang L. (2017). Identification, and biocontrol potential of antagonistic bacteria strains against *Sclerotinia sclerotiorum* and their growth-promoting effects on *Brassica napus*. Biol. Control..

[52] Zhou H., Ren Z.H., Zu X., Yu X.Y., Zhu H.J., Li X.J., Zhong J., Liu E.M. (2021). Efficacy of Plant Growth-Promoting Bacteria *Bacillus cereus* YN917 for Biocontrol of Rice Blast. Front Microbiol..

[53] Myo E.M., Liu B., Ma J., Shi L., Jiang M., Zhang K., Ge B. (2019). Evaluation of *Bacillus velezensis* NKG-2 for bio-control activities against fungal diseases and potential plant growth promotion. Biol. Control..

[54] Wu H., Wu Q., Wu G., Gu Q., Wei L. (2016). Cd-Resistant Strains of *B. cereus* S5 with Endurance Capacity and Their Capacities for Cadmium Removal from Cadmium-Polluted Water. PLoS ONE.

